# Notes and News

**Published:** 1898-04-16

**Authors:** 


### Notes and News.

(Collected and Communicated.)

Mr. Hope Morley, treasurer of the Royal Hospital for Diseases of the Chest, City Road, has contributed £500 to the Festival Dinner Fund.

The *New Century Review* for April contains an interesting article by Mr. Kineton Parks on "Lead Poisoning in the Potteries."

Lady Glenesk will be much missed at the Chelsea Hospital for Women, an institution in which she was much interested.

We are glad to note that 80 beds are now open at the City of London Hospital for Diseases of the Chest. Through a mistake, we mentioned 65 as being the number last week.

The Royal gifts to the Netley Hospital have been received with much enthusiasm, owing to the graceful form these mementoes of the Queen's visit have taken. Each ward now boasts the latest photograph of Her Majesty, signed with her autograph. In addition to these, the Queen has presented the hospital with a portrait of the Prince Consort, and engravings of several members of the Royal family. She has also given instructions that those soldiers whom she found on the occasion of her visit minus a leg or arm are to be provided with the most efficient artificial limbs it is possible to procure. Princess Henry of Battenberg has presented a steel engraving portrait of the late Prince Henry, this being inscribed: "A gift of a soldier's widow to soldiers who have fought and bled for their country."

The evidence given at an inquest recently held at Sydenham shows how easy it is for the public to obtain access to the most virulent poisons, notwithstanding all the regulations by which their sale is thought to be safeguarded. The inquest in question was held on the body of Eleanor Marx, otherwise known as Eleanor Marx Aveling, who had lived for the past fifteen years as the wife of Dr. Aveling, lecturer. According to the evidence of the servant, the deceased sent her to a chemist's shop with a note, and she brought back a small parcel and a book to be signed. Soon afterwards she found her mistress undressed in bed and dying. The chemist said that the note was for enough prussic acid to kill a dog, and he sent it under the impression that the note was signed by Dr. Aveling, and that he was a qualified medical man. Be this as it may, it seems fairly clear that, if a person contemplates murder or suicide, it is hardly likely that he will draw the line at forgery, and that the mere, unwitnessed, signing of someone else's name in a book will not stand greatly in the way of his obtaining possession of any form of poison he may require.

Sir Samuel Wilks has been re-elected President of the Royal College of Physicians.

At Middlesbrough crops of small-pox cases continue to be reported from time to time.

The refusal of the Local Government Board to sanction any more loans on the part of the Guardians, for the Selby Oak Infirmary, has aroused much local interest in regard to the extravagant and unusual amount the Guardians proposed to expend on this infirmary.

Cases of small-pox are reported from Newcastle-on-Tyne and Gateshead, so we may expect before long to see a considerable development of the disease in the North. Middlesbrough was too large a centre to be infected for long without doing injury to its neighbours.

The *New Century Review* has a good article by Mr. Escott on "Army Doctors Up to Date," in which the absurdity of the present position is well shown up. He points out with great force that the real obstacle to the only practicable solution of the problem of providing doctors for the army lies not with Lord Lansdowne, but with his military advisers, and, above all, with those obstinate incarnations of British militarism who, because of the admiration which their exploits in the field compelled, are mistakenly allowed to assert a power which is wholly mischievous in the council. "*Fou comme un vieu militaire* is a proverb whose truth has long been practically recognised in France, and the ignoring of which cannot be due to lack of instances of its applicability to England."

In addition to the usual courses of First Aid to the Injured which are given periodically at the Seamen's Mission at Poplar, a special series has lately been given for the purpose of instructing sea going crews in the simple elements of sick-nursing. The making of poultices, the application and administration of simple remedies, lotions, &c., has been included, together with the most valuable hints as to the care of the health in tropical climates. Dr. Godding, the lecturer, is most popular at the institute, and his classes of sailors thoroughly appreciate the admirable hints he gives them on many little simple remedial agents which should find their way into the sailor's sea-chest. This kind of instruction for sailors is a new departure, and has proved so successful that it will probably form an integral part of the first aid instruction given at the institute.

Truly, the medical details of the popular novel frequently take a most amusing form. One such, written by a woman novelist—which has attained a high degree of success—gives a dramatic account of the symptoms following on the drinking of nearly a tumblerful of chloral solution, surely a larger dose than is necessary even for the most sensational situation. In five minutes "the coffee-brown eyes had dwindled to two hard dots." The person who had taken the chloral was an invalid, who for nine years had not set foot to the ground. Notwithstanding this, so soon as she discovered the poison she had taken, which was within a minute of swallowing it, "she tore and dragged and staggered up and down her room for three whole hours," and ultimately died a dramatic death. It would have been so much more simple to take an emetic and live. Clearly the author confuses opium and chloral.

